# Cardiac T1 Mapping and Extracellular Volume (ECV) in clinical practice: a comprehensive review

**DOI:** 10.1186/s12968-016-0308-4

**Published:** 2016-11-30

**Authors:** Philip Haaf, Pankaj Garg, Daniel R. Messroghli, David A. Broadbent, John P. Greenwood, Sven Plein

**Affiliations:** 1 Division of Biomedical Imaging, Leeds Institute of Cardiovascular and Metabolic Medicine (LICAMM), University of Leeds, Leeds, LS2 9JT UK; 2 Department of Cardiology and Cardiovascular Research Institute Basel (CRIB), University Hospital Basel, Petersgraben 4, 4054 Basel, Switzerland; 3 Department of Internal Medicine – Cardiology, German Heart Institute Berlin, Berlin, Germany

**Keywords:** T1 mapping, ECV, Cardiomyopathy, Acute chest pain syndromes, Diffuse myocardial fibrosis

## Abstract

Cardiovascular Magnetic Resonance is increasingly used to differentiate the aetiology of cardiomyopathies. Late Gadolinium Enhancement (LGE) is the reference standard for non-invasive imaging of myocardial scar and *focal* fibrosis and is valuable in the differential diagnosis of ischaemic versus non-ischaemic cardiomyopathy. *Diffuse* fibrosis may go undetected on LGE imaging. Tissue characterisation with parametric mapping methods has the potential to detect and quantify both focal and diffuse alterations in myocardial structure not assessable by LGE. Native and post-contrast T1 mapping in particular has shown promise as a novel biomarker to support diagnostic, therapeutic and prognostic decision making in ischaemic and non-ischaemic cardiomyopathies as well as in patients with acute chest pain syndromes. Furthermore, changes in the myocardium over time may be assessed longitudinally with this non-invasive tissue characterisation method.

## Background

Cardiovascular Magnetic Resonance (CMR) is increasingly used to differentiate the aetiology of cardiomyopathies. Its three-dimensional nature with excellent spatial resolution and high tissue contrast enables accurate measurement of *cardiac function and morphology*: left ventricular volumes, mass and ejection fraction as well as an assessment of regional wall motion abnormalities can be achieved largely independent of body habitus, imaging windows and without ionising radiation exposure [1]. Recent advances in CMR provide the potential to also assess and quantify *myocardial tissue composition* [2]. This article aims to review and illustrate advances in parametric mapping methods, in particular T1 mapping in cardiac diseases and to appraise their clinical potential in the context of established CMR methods.

## Late gadolinium enhancement

Late Gadolinium Enhancement (LGE) has become the reference standard for non-invasive imaging of myocardial scar and focal fibrosis in both ischaemic [3] and non-ischaemic cardiomyopathy [4]. LGE imaging depicts the relative difference in longitudinal recovery times (T1) between enhancing areas of fibrosis or scar (T1 shortened due to accumulation of extracellular gadolinium contrast agent) and normal nulled myocardium (longer T1 as gadolinium contrast agent is more rapidly washed out) [2]. The method has particular value in the differential diagnosis of ischaemic versus non-ischaemic cardiomyopathy based on the location and transmural extent of scar. Based upon specific LGE patterns some of the non-ischaemic cardiomyopathies can be further differentiated. Diffuse fibrosis can go undetected on LGE imaging because of the absence of normal reference myocardium and the identification of microscopic interstitial fibrosis is limited by the spatial resolution of LGE images. In the setting of diffuse fibrosis, presence of LGE has been shown to correlate poorly with collagen volume calculated from endomyocardial biopsies [2]. Although numerous quantification methods for LGE exist, the presence of fibrosis and scarring is generally identified qualitatively by visual interpretation of LGE images, limiting the ability to compare findings between subjects or in follow-up examinations.

## Principles of T1 mapping

**Fig. 1:**
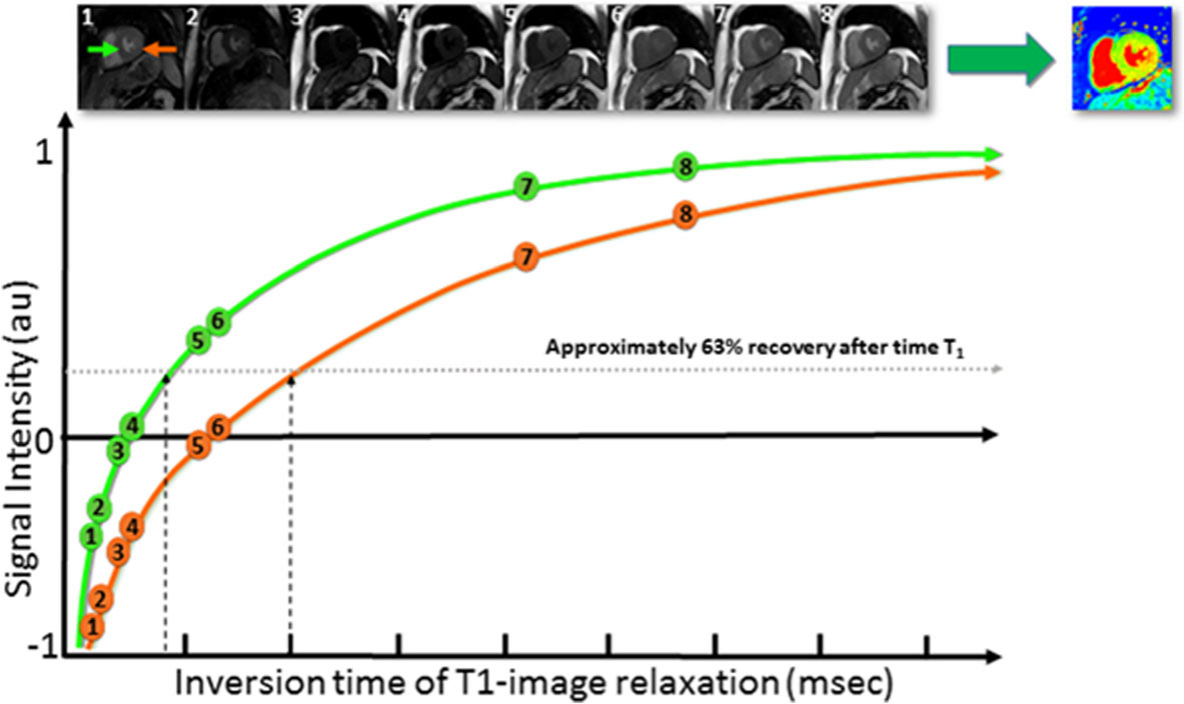
Modified Look-Locker Inversion Recovery (MOLLI) scheme for T1-mapping in the heart. This protocol employs two inversions to acquire eight images over 11 heart beats, referred to here as 5(3)3, which means five images are acquired over consecutive cardiac cycles followed by a three heart beat gap and then three images are acquired over consecutive cardiac cycles. 5s(3s)3s MOLLI schemes would acquire images for a duration of 5s followed by a gap of 3s and a second acquisition train lasting 3s, further minimizing heart rate dependency of the results. For illustrative purpose, the *orange arrow* and relaxation curve refer to an area of myocardial infarction and elevated native T1 values. The *green arrow* and relaxation curve refer to an area of normal septal myocardium and normal native T1 values. Images are sorted by inversion times

T1 mapping measures the longitudinal or spin-lattice relaxation time, which is determined by how rapidly protons re-equilibrate their spins after being excited by a radiofrequency pulse. In 1970, Look and Locker proposed methods to measure T1 relaxation times by acquiring data successively after magnetisation inversion [5]. Subsequently, these methods have been refined and acquisition times shortened. The Modified Look-Locker Inversion recovery (MOLLI) pulse sequence allows measurement of T1 times in a single breath hold over 17 successive heart beats and has become the most popular T1 mapping method [6]. The main difference between conventional Look-Locker and MOLLI is that in the latter the images are acquired at the same cardiac phase allowing mapping. Variations of MOLLI have been proposed allowing shortened breath-hold durations and reduced sensitivity to heart rate, such as the 5(3)3 scheme indicated in Fig. 1. The Shortened MOLLI (ShMOLLI) scheme uses sequential inversion-recovery measurements with a single breath hold of only nine successive heart beats [7] and a conditional fitting algorithm to account for the short recovery period between inversion pulses. Other pulse sequences including saturation recovery single-shot acquisition (SASHA) [8] and saturation pulse prepared heart-rate-independent inversion recovery (SAPPHIRE) [9] are also used in clinical practice.

T1 mapping refers to pixelwise illustrations of absolute T1 relaxation times on a map. T1 mapping circumvents the influence of windowing and nulling (as in LGE) and allows direct T1 quantification. As such, T1 mapping has the potential to detect diffuse myocardial structural alterations not assessable by other non-invasive means, including LGE.

Currently used T1 mapping methods acquire a set of non-segmented raw images within separate cardiac cycles of a single breath-hold. As a result, the acquisition duration for each raw image is limited to approximately 200 ms within the cardiac cycle, which limits the spatial resolution that can be achieved. In addition, poor breath-holding can significantly impair the quality of T1 maps, which can be compensated for to some extent by the application of manual or automatic motion correction. The differences in acquisition schemes have a direct effect on the range of normal and abnormal T1 with a given technique [10], which means that absolute T1 values can only be directly compared when they were obtained with the same acquisition scheme at the same field strength using the same post-processing methods. Thus, reports on T1 values should always include the T1 mapping technique that was used and the site-specific normal range for T1 [11]. Motion correction is essential for high quality T1 mapping and is generally achieved with breath holding. Image quality can be improved with respiratory motion compensation methods in patients with poor breath-holding [12] and phase sensitive inversion recovery reconstruction [13] further improved image quality. Nevertheless, residual uncorrected respiratory motion is still problematic particularly if unrecognized and in areas of thin myocardium [14].

## Native T1 mapping

Native T1 values are primarily influenced by the field strength used, with higher native T1 values at 3 T than at 1.5 T [15]. Measured T1 values also depend on the pulse sequence used (MOLLI and ShMOLLI generally underestimate T1), the cardiac phase (diastole versus systole) and region of measurement [15]. Normal native T1 values are thus specific to the local set-up [16] and need to be reassessed when the acquisition method is changed. Any of the currently used pulse sequence schemes have demonstrated very high inter-study reproducibility for native myocardial T1.

The two most important biological determinants of an increase in native T1 are *oedema* (increase of tissue water in e.g. acute infarction of inflammation) and increase of *interstitial space* (e.g. fibrosis of infarction (scar) or cardiomyopathy, and in amyloid deposition). The two most important determinants of low native T1 values are *lipid* overload (e.g. Anderson-Fabry disease, lipomatous metaplasia in chronic myocardial infarction) and *iron* overload. Native T1 values are a composite signal of myocytes and extracellular volume (ECV) with the potential of pseudonormalization of abnormal values (e.g. low native T1 values of Anderson-Fabry disease cancelled out by inferolateral fibrosis). Native T1 mapping is feasible even in patients with severe renal impairment in whom gadolinium-based contrast agents are contraindicated.

## Contrast-enhanced T1 Mapping and Extracellular Volume (ECV) fraction

Contrast-enhanced T1 mapping is used for mostly calculating the ECV fraction in combination with native T1 mapping. Standard gadolinium-based contrast agents are distributed throughout the extracellular space and shorten T1 relaxation times of myocardium proportional to the local concentration of gadolinium [2]. Areas of fibrosis and scar will therefore exhibit shorter T1 relaxation times, in particular after contrast administration. The haematocrit represents the cellular fraction of blood. Estimation of the ECV (interstitium and extracellular matrix) requires measurement of myocardial and blood T1 before and after administration of contrast agents as well as the patient’s haematocrit value according to the formula:

$$ ECV=\left(1- haematocrit\right)\frac{\frac{1}{post\  contrast\ T1\ myo} - \frac{1}{native\ T1\ myo}}{\frac{1}{post\  contrast\ T1\  blood} - \frac{1}{native\ T1\  blood}} $$

**Fig. 2:**
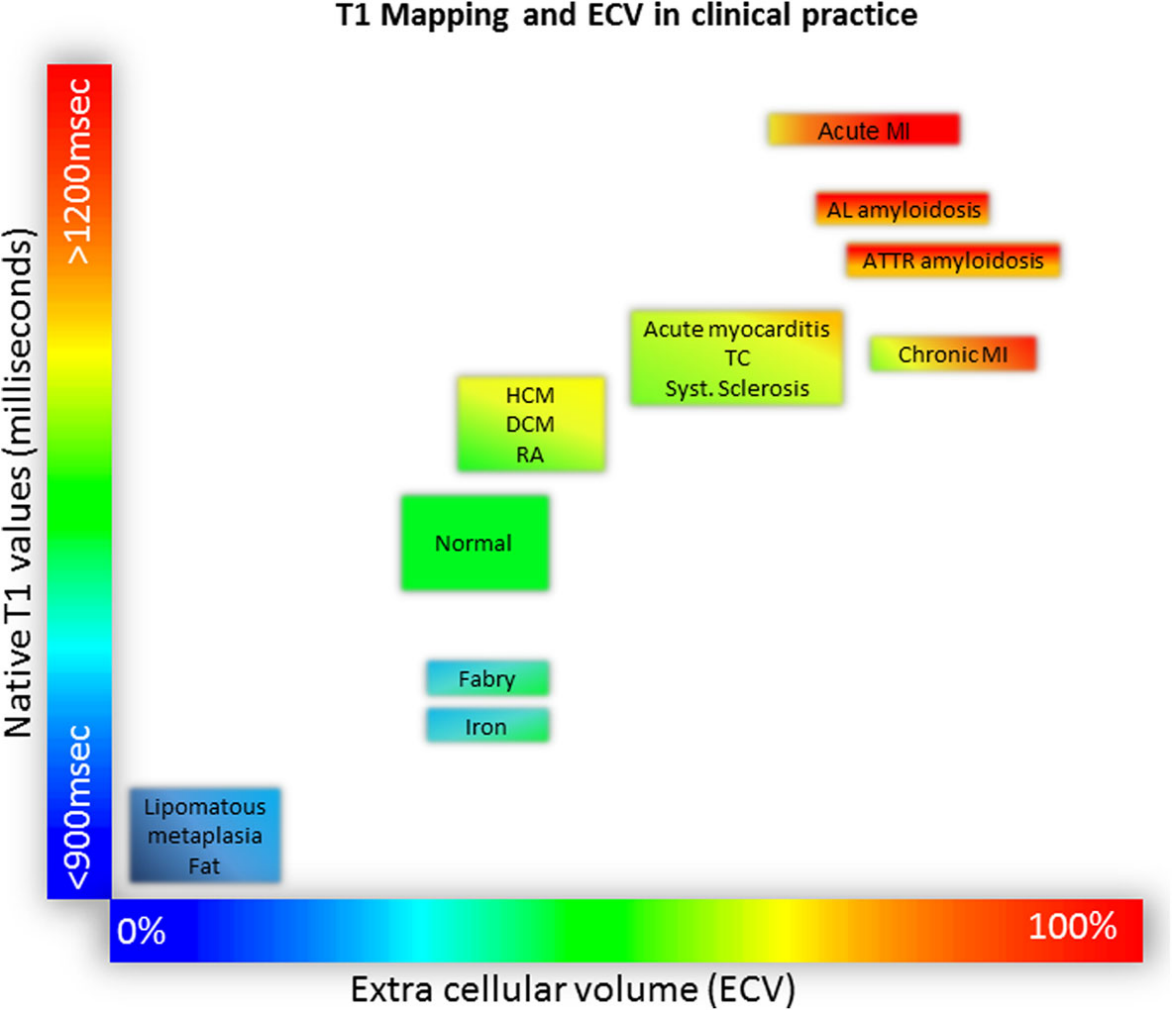
Tissue characterisation using native T1 and extracellular volume fraction (ECV). Absolute values for native T1 depend greatly on field strength (1.5 T or 3 T), pulse sequence (MOLLI or ShMOLLI), scanner manufacturer and rules of measurements. For the purpose of comparability, only studies using 1.5 T scanners were considered in this figure. Figure adapted from Martin Ugander (SCMR 2014)

ECV is a marker of myocardial tissue remodelling and provides a physiologically intuitive unit of measurement. Normal ECV values of 25.3 ± 3.5% [1.5 T] have been reported in healthy individuals [17] (Fig. 2). Apart from *amyloid*, an increased ECV is most often due to excessive *collagen* deposition and is thus a more robust measure of myocardial fibrosis. Low ECV values occur in *thrombus* and *fat/lipomatous metaplasia*. ECV can either be calculated for myocardial regions-of-interest or visualized on ECV maps.

Unlike native T1 relaxation times, contrast-enhanced T1 values are more variable and dependent on contrast agent dosing, the time elapsed between contrast agent administration and T1 measurement and renal clearance. ECV on the other hand represents a physiological parameter and is derived from the ratio of T1 signal values. ECV values may therefore be more reproducible between different field strengths, vendors and acquisition techniques than both native and post-contrast T1 [11]. ECV measures also exhibit better agreement with histological measures of the collagen volume fraction than isolated post-contrast T1 [18].

## Clinical use of T1 mapping and ECV

### Acute chest pain syndromes

**Fig. 3:**
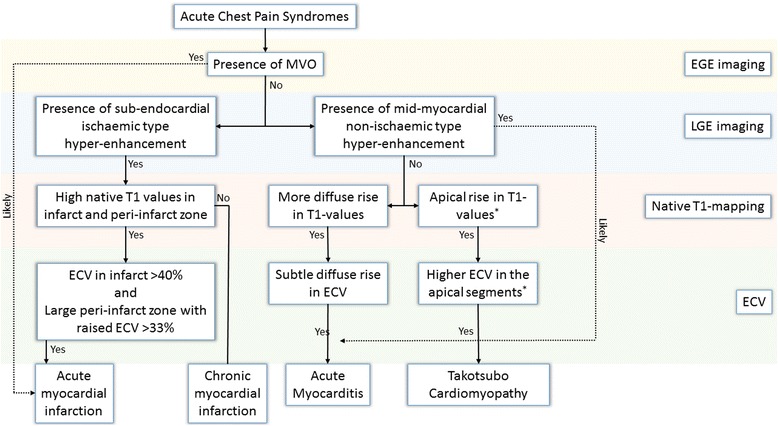
Acute chest pain syndromes algorithm using multi-parametric tissue characterisation. ECV denotes extra-cellular volume, LGE Late Gadolinium Enhancement, and MVO microvascular obstruction. . *This holds true for classical type 1 Takotsubo Cardiomyopathy

Native T1 and ECV help in the differential diagnosis of patients with acute chest pain including acute coronary syndrome, myocarditis and Takotsubo cardiomyopathy and can help in the distinction of acute from chronic infarction (Fig. 3).

### Acute myocarditis

**Fig. 4:**
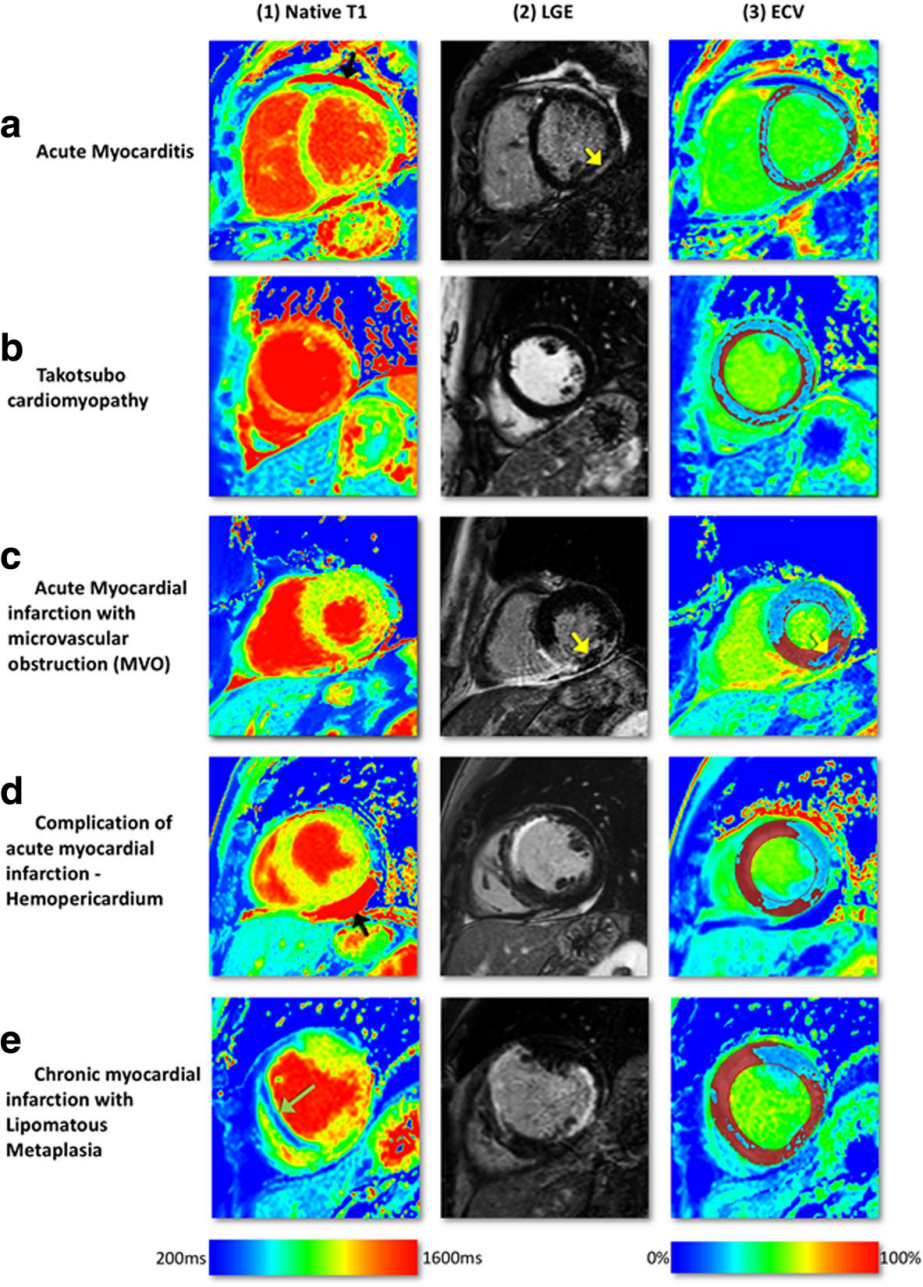
Multi-parametric tissue characterisation at mid-slice in acute chest pain syndromes. On ECV-maps, *red* areas represent ECV greater than 30%. T1-mapping was done using a modified Look-Locker Inversion Recovery (MOLLI) pulse sequence on 1.5 Tesla Ingenia, Philips, Best, The Netherlands. **a** Acute myocarditis with higher native T1-values in the infero-lateral wall of the left ventricle (a1) consistent with LGE in the mid inferior-lateral wall (a2, *yellow arrow*). The ECV map (a3) demonstrates diffusely increased extra-cellular space. **b** Takotsubo Cardiomyopathy (TC) with diffusely high native T1 values (b1), no evidence of focal LGE (b2) and diffusely increased ECV (b3). **c** Acute re-perfused ST-elevation myocardial infarction affecting the inferior wall. Native T1-vales are raised in the area of risk (>1000ms) and also in the remote myocardium. On LGE imaging, inferior infarction with an area of microvascular obstruction can be seen (*yellow arrow*, c2). ECV is raised in the infarct zone but low in the MVO as this area does not take up any contract (*yellow arrow*, c3). **d** Anterior wall ST-elevation myocardial infarction with rupture of the left ventricle free wall (not seen in these images) resulting in haemo-pericardium. The pericardial haemorrhage has high native T1 values (*black arrow*, d1), high signal on LGE and low ECV values (d3). **e** Chronic MI in the antero-septal wall. There is an area of reduced native T1 values in the septum (*green arrow*, e1) which corresponds to lipomatous metaplasia transformation in previous antero-septal infarct. There is also an acute infarction in the lateral wall with some peri-infarct oedema seen on native T1. Abbreviations: AMI, acute myocardial infarction; ECV, extra-cellular volume; MI, myocardial infarction; LGE, Late Gadolinium Enhancement; TC, Takotsubo Cardiomyopathy

Endomyocardial biopsy is still the gold standard for confirmation of myocarditis but remains limited by frequent sampling errors reducing diagnostic yield and its invasiveness. In clinical practice, clinical history, laboratory analyses and imaging findings are therefore generally used to diagnose acute myocarditis. The “Lake-Louise” CMR [19] criteria have been widely used to diagnose myocarditis: the diagnosis is likely if two of the three criteria *myocardial oedema* (T2-weighted imaging), *LGE in a mid-wall non-coronary pattern often in the infero-lateral wall*, and *hyperaemia/capillary leak* (increased early gadolinium enhancement ratio between myocardium and skeletal muscle) are present. Radunski et al. have performed a comprehensive comparison of the diagnostic accuracy of conventional CMR techniques and novel mapping techniques and demonstrated better diagnostic accuracy of T1 mapping and in particular by ECV [20] (Fig. 4a). Both T1 and ECV mapping allow for more sensitive identification and quantification of diffuse myocardial fibrosis and oedema than LGE. LGE together with ECV quantification (ECV ≥27% as diagnostic criterion) significantly improved the diagnostic accuracy to 90% (95% CI: 84–95%) compared with 79% (95% CI: 71–85%; *p* = 0.0043) for the “Lake-Louise” CMR criteria [19]. In patients with severe myocarditis (new-onset heart failure or acute chest pain) raised native T1 (1098 ± 41 ms [1.5T]) and ECV (31 ± 3% [1.5T]) have been reported [20] (Fig. 2). High diagnostic performance (~90% overall sensitivity, specificity and diagnostic accuracy) has been reported for detecting changes in myocarditis using an absolute T1 cut-off of 990 ms [21]. The cut-off value proposed in this study is however specific to the field strength, vendor and T1 mapping technique used and cannot be universally applied.

### Takotsubo cardiomyopathy

Acute but rapidly reversible mid and apical left ventricular (LV) segment akinesia with ballooning and compensatory hyperkinesia of basal segments is the typical finding in Takotsubo or stress-induced cardiomyopathy. Typically there are no perfusion defects and no scar on LGE imaging in contrast to myocarditis and myocardial infarction. In current clinical practice, T2-weighted imaging using the short-tau inversion recovery (STIR) sequence is used to detect oedema. T1 mapping has potential advantages over T2 STIR as it is a quantitative method that is not in need of a reference region of interest (ROI) and it can be obtained in a single breath hold. Ferreira et al. demonstrated elevated native T1 values with a good correlation between T1 values and T2 signal intensity (SI) ratios and high diagnostic accuracy (AUC = 0.94, sensitivity and specificity of 92%) in the differentiation between oedema and normal myocardium [22] (Figs. 2 and 4b). Elevated native T1 values can both be caused by myocardial oedema and fibrosis. In clinical practice, a presumable myocardial oedema zone in Takotsubo cardiomyopathy might be further substantiated with T2-weighted imaging such as STIR sequences or T2 mapping sequences. The presence of fibrosis, as may exist if the patient has another underlying pathology, may cause an increased ECV.

### Acute myocardial infarction

Ischaemia triggers the development of cellular oedema. Native T1 reliably detects segmental abnormalities caused by acute myocardial infarction (MI) with high sensitivity and specificity [23]. T1 mapping detects myocardial oedema in both ST-elevation MI (STEMI) and non ST-elevation MI (NSTEMI) patients [24] and is at least as sensitive as T2-STIR [22], in particular in patients with smaller infarcts [23] (Fig. 4c, d). Although the distinction of acute vs. chronic MI can be challenging, T1 values in acute MI are generally higher than in chronic MI and thus may allow distinction of an acute coronary syndrome (ACS) from chronic injury. Prescribing a distinct cut-off value that can be used in an individual patient is hampered by the general variability of native T1 values between subjects, the influence of field strength and acquisition pulse sequence on T1 values and the influence of infarct size on T1 values. In practice, the distinction of acute vs. chronic MI remains mainly based on the overall assessment of infarct, oedema (area at risk), and microvascular obstruction zone.

Furthermore, T1 values progress from normal myocardium to that of maximal injury and can be used for defining the peri-infarct zone/area-at-risk [24]. The longitudinal relaxation time measured by T1 mapping is mainly related to tissue fibrosis and oedema. Carrick et al. [25] have shown that an infarct core with native T1 values lower than the surrounding area at risk correlated with the microvascular obstruction zone by contrast-enhanced CMR and was associated with worse clinical outcome.

A recent study of 300 patients with reperfused STEMI demonstrated native T1 remote from infarcted myocardium at baseline to be independently predictive of adverse LV remodelling and adverse cardiac events 6 months post-STEMI [26]. Native T1 values in acute MI are high and ECV values are among the highest of all cardiac disease (58.5 ± 7.6) [17], most likely due to disruption of cardiomyocyte membrane integrity and subsequent expansion of the distribution volume of extracellular contrast agents (Fig. 2).

Microvascular obstruction in the infarct core (no-reflow phenomenon) results in a pseudo-normalization of T1 values in this area [23, 24]. Due to accumulation of methaemoglobin (T1 shortening effect), T1 can even be decreased in the case of intramyocardial haemorrhage (Fig. 4c).

Native T1 mapping might also be useful in the assessment of complication of AMI as illustrated in Fig. 4d showing haemo-pericardium with high native T1 values in a patient with ruptured LV free wall.

### Chronic myocardial infarction

In chronic MI, the necrotic and oedematous infarct tissue of an acute infarct is replaced by a smaller area of increased extracellular collagen (fibrous scar). Native T1 values are therefore lower and less extensive in chronic MI compared with the acute stage. The ECV of chronically infarcted myocardium has been shown to be markedly elevated (51 ± 8%) compared to normal myocardium but slightly lower than in acutely infarcted myocardium (Figs. 2 and 4e) [27].

T1 mapping is also able to illustrate areas of lipomatous metaplasia in chronic MI, the presence of which alters the electrical properties of the myocardium and might play a role in post-MI arrhythmogenesis [28]. Fat has very low T1 values (230–350 ms at 1.5 T) [29] and the fatty replacement area within the infarct core therefore displays noticeable T1 decrease [24].

## Non-ischaemic cardiomyopathies

### Cardiac amyloidosis

Amyloidosis can be regarded as the exemplar of an interstitial disease. Although endomyocardial biopsy remains the reference standard for diagnosis, it is not routinely performed because it is invasive and prone to sampling errors with false-negative results. The typical constellation on echocardiography (concentric LV hypertrophy, bi-atrial dilatation, restrictive filling), ECG (low-voltage QRS in spite of LV hypertrophy) and elevated blood biomarkers (cardiac troponin and natriuretic peptides) is found mainly in advanced disease. Myocardial amyloid deposition results in interstitial expansion, which can be visualized by typically patchy or subendocardial LGE with early blood pool darkening on Look Locker scout images. The characteristic diffuse LGE enhancement though makes nulling of normal myocardium particularly difficult, often leading to confusion in interpretation [30].

**Fig. 5:**
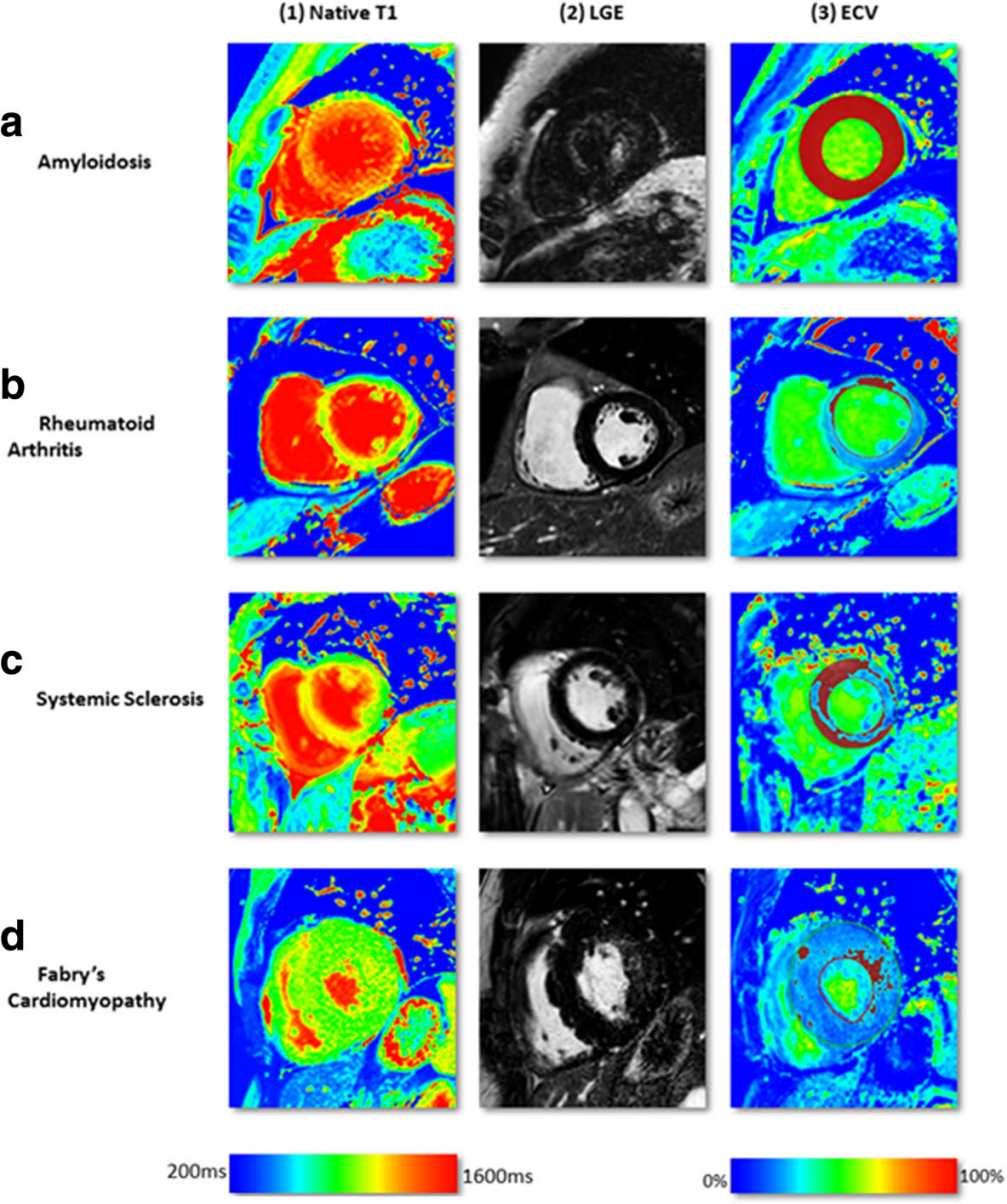
Multi-parametric tissue characterisation at mid-slice in diseases involving myocardium. On ECV-maps, red areas represent ECV greater than 30%. T1-mapping was done using a modified Look-Locker Inversion Recovery (MOLLI) pulse sequence on 1.5 Tesla Ingenia, Philips, Best, The Netherlands. **a** Biopsy proven cardiac amyloidosis. T1 maps show diffuse rise in native-T1 values (a1). On LGE-imaging, there is low contrast-noise ratio (CNR) between the blood pool and the myocardium (a2). ECV-maps demonstrate diffuse rise in extra-cellular space in the whole myocardium. **b** Established rheumatoid arthritis demonstrating some rise in native T1 (b1) and ECV (b3) with normal signal distribution on LGE-imaging (b2). **c** Established Systemic Sclerosis demonstrating rise in native T1 values predominantly in the septum (c1) and more widespread increase in ECV (c3). There is no evidence of any scar or fibrosis on LGE-imaging. **d** Bio-chemical diagnosis of Fabry’s disease: Native T1 (e1) demonstrates pseudo-normalization due to the effects of replacement fibrosis exceeding the fatty-related T1 decrease. LGE (e2) demonstrates fibrosis of the lateral wall in consistence with the ECV map (e3). Abbreviations: ECV, extra-cellular volume; LGE, Late Gadolinium Enhancement

T1 mapping circumvents the limitations of myocardial nulling faced in LGE imaging, provides quantitative assessment of diffuse extracellular expansion, and is a viable option in renal failure, which is common with amyloid. Performed serially, it might be a means to follow response to treatment and changes in myocardial burden [31]. Both types of cardiac amyloidosis show markedly elevated native T1 values (Figs. 2 and 5a). Using native T1 cardiac amyloidosis could be reliably diagnosed and differentiated from hypertrophic cardiomyopathy, a clinically relevant differential diagnosis [32].

Cardiac amyloid is associated with a higher ECV than any other cardiomyopathy (ECV 46.6 ± 7.0%) due to the widespread and substantial extracellular infiltration [17].

Early detection of cardiac amyloidosis and differentiation between the two main forms *transthyretin-related cardiac amyloidosis* (ATTR) and *light chains cardiac amyloidosis* (AL) is of high clinical importance because untreated cardiac amyloidosis has poor prognosis. In addition to supportive heart failure therapy, specific treatment options are available for both ATTR (liver transplantation, novel TTR-specific treatment) and AL (chemotherapy, autologous stem cell transplantation) [33]. Efforts to differentiate between ATTR and AL have been made by Dungu et al., who reported higher LV mass in patients with ATTR compared to AL and proposed a sum LGE score (QALE score) for differential diagnosis [34]: LGE patterns seem to be more extensive, diffuse and transmural in ATTR (QALE score ≥13) and more often showing a less extensive, more subendocardial pattern in AL cardiac amyloidosis (QALE score <13) [34]. The overlap between AL and ATTR amyloidosis, though, remains substantial. Given the great therapeutic consequence of ATTR (liver transplantation, novel TTR-specific treatment) vs. AL amyloidosis (chemotherapy, autologous stem cell transplantation) currently further testing is required with cardiac biopsy, genetic testing, or nuclear scanning to confidently distinguish between the two disease types.

Nevertheless ECV has been proposed to become the first non-invasive test to quantify cardiac amyloid burden and could be used as a tool to guide and monitor treatment [35].

## Systemic cardiac disorders

### Rheumatoid arthritis

Subclinical cardiovascular disease is common in patients with rheumatoid arthritis (RA) and predominantly affects young female subjects. Up to 39% of RA patients have been reported to show focal LGE patterns, probably related to earlier myocarditis [36]. However, diffuse fibrosis is common in RA and cannot be reliably detected by LGE. In a pilot study, native T1 values were slightly elevated in RA patients compared to controls and RA patients had expanded ECV (30.3 ± 3.4 vs. 27.9 ± 2.0; *p* < 0.001) [37] (Figs. 2 and 5b). Disease activity scores correlated with diffuse fibrosis and systolic and diastolic strain regardless of LGE [37].

### Systemic sclerosis

Cardiac involvement is common in systemic sclerosis, often before cardiac symptoms occur. Low grade inflammation and diffuse myocardial fibrosis are well-described co-existing disease processes in systemic sclerosis and can be detected by T2-STIR and LGE imaging. As in other diseases, LGE is limited in the assessment of diffuse myocardial fibrosis, especially when the entire myocardium may be affected more homogeneously as occurs with systemic sclerosis. In a small study, native T1 and ECV (35.4 ± 4.8 vs. 27.6 ± 2.5%) were elevated in patients with systemic sclerosis (Figs. 2 and 5c) [38].

### Anderson-fabry disease

Anderson-Fabry disease (AFD) is an intracellular lipid disorder (lysosomal storage disease) that causes concentric LV hypertrophy, heart failure and arrhythmias [39].

On LGE images, AFD typically displays an infero-lateral mid-wall pattern of enhancement caused by focal fibrosis in this region. In addition, the low native T1 of fat can serve as an early surrogate marker of myocardial glycosphingolipid storage in AFD even before the development of LV hypertrophy [40]. Native T1 reliably distinguished AFD from other common causes of LV hypertrophy using a predefined cut-off [40]. However, segmental T1 analysis in the infero-lateral wall showed pseudo-normalized or even elevated T1 due to the effects of replacement fibrosis exceeding the fatty-related T1 decrease [40]. Unlike native T1, the ECV in AFD is typically normal as AFD is an *intra*cellular (lysosomal) storage disease [17] and ECV values in AFD have been reported to be similar to healthy controls (ECV 21.7 ± 3.0% [1.5T]) [41] (Figs. 2 and 5d).

### Iron-overload cardiomyopathy

Iron-overload develops primarily from increased absorption such as in genetic hemochromatosis or secondary to repeated blood transfusions, as in thalassaemia major [42]. Cardiac iron deposition confers a poor prognosis without (chelation) therapy [42]. Iron as a ferromagnetic material is known to shorten the three fundamental tissue MRI signal constants, T1, T2 and T2*. T2* currently is the non-invasive gold standard method to quantify iron deposition in myocardium [43]. Sado et al. have shown shown that native T1 values were lower in patients with iron-overload cardiomyopathy with good correlation with T2* [44] (Fig. 2). Compared with T2* mapping, T1 has the advantage of higher reproducibility, easier clinical use with less offline analysis needed and the potential to detect early iron overload [44]. On the other hand, unlike T2*, T1 is less pathologically specific and *increased* in various other cardiomyopathies involving increased interstitial space (fibrosis, amyloid). Therefore, early iron deposition may be missed in these patients.

## Diffuse fibrosis

### Hypertrophic cardiomyopathy

**Fig. 6:**
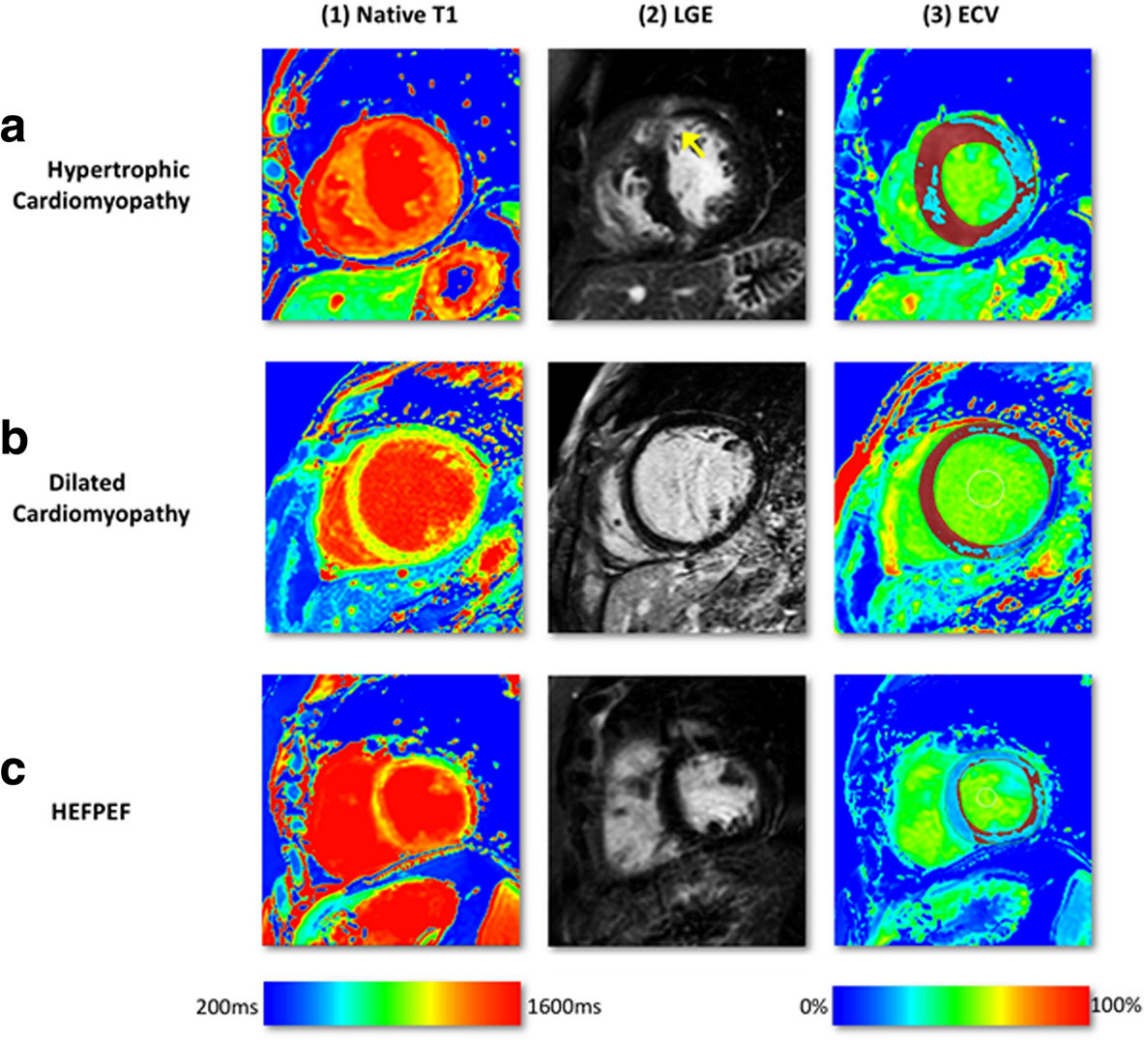
Multi-parametric tissue characterisation at mid-slice in cardiomyopathies. On ECV-maps, *red areas* represent ECV greater than 30%. T1-mapping was done using a modified Look-Locker Inversion Recovery (MOLLI) pulse sequence on 1.5 Tesla Ingenia, Philips, Best, The Netherlands. **a** HCM showing diffuse and heterogeneous LGE in the anterior wall (*yellow arrow*, a2). Native T1 was diffusely raised, exceeding the hypertrophied segments (a1). ECV-maps demonstrate higher ECV in and around the diffuse LGE (a3). **b** DCM with no LGE enhancement (b2) but raised native T1 values in the septum (1000–1200ms) (b1) and raised ECV (b3). **c** HFpEF Native-T1 values were significantly raised through-out (>1000ms) with no presence of scar on LGE-imaging (c2). ECV maps demonstrated patchy rise in extra-cellular space (c3). Abbreviations: DCM, dilated cardiomyopathy; ECV, extra-cellular volume; HFpEF, heart failure with preserved ejection fraction; HCM, hypertrophic cardiomyopathy; LGE, Late Gadolinium Enhancement

Autosomal dominant mutations involving sarcomeric genes lead to hypertrophic cardiomyopathy (HCM) and a combination of myocyte disarray, fibrosis and ventricular hypertrophy in distinct patterns [45]. Clinically, HCM is diagnosed by a combination of history (pedigree), ECG signs and an evaluation of LV wall thickness. LGE typically occurs at right ventricular (RV) insertion points and with variable frequency and severity in hypertrophied, often hypocontractile segments [45]. Histologically, fibrosis is often more global, or diffuse, and often undetectable by standard LGE pulse sequences (nulled reference tissue potentially in area of diffuse fibrosis). Native T1 values are prolonged in HCM and correlate with wall thickness suggesting that it is a marker of disease severity [46, 47]. Patients with HCM have reduced post-contrast myocardial T1 consistent with the presence of diffuse interstitial fibrosis outside areas of LGE. ECV in HCM (29.1 ± 0.5% [1.5T]) [17] in segments without LGE has shown to be in the upper normal range of normal patients (Figs. 2 and 6a) [48]. ECV can be used in the differential diagnosis of HCM vs. athletic remodelling in athlete’s heart, in particular in those subjects in the grey zone of LV wall thickness (12–15 mm). Whereas ECV increases with increasing LV hypertrophy in HCM (due to extracellular matrix expansion and myocardial disarray), ECV reduces in athletes with increasing wall thickness (due to an increase in healthy myocardium by cellular hypertrophy) [48]. The impact of myocardial disarray on T1 mapping in HCM, though, remains controversial and may result in overestimation of ECV [49].

### Dilated cardiomyopathy

LV or biventricular dilatation and systolic dysfunction without an obvious or detectable cause are the defining characteristics of dilated (nonischaemic) cardiomyopathy (DCM). In DCM, LGE typically occurs in a mid-wall pattern [50] but in the majority of DCM there is lack of any detectable LGE. Native T1 values are prolonged in DCM and correlate with reduced wall thickness [46, 47]. ECV measurement reflects myocardial collagen content in DCM and might serve as a non-invasive imaging biomarker to monitor therapy response and aid risk stratification in different stages of DCM [51]. ECV in DCM has been shown to be in a similar range to HCM (28 ± 0.4% [1.5T]) [17] (Figs. 2 and 6b). The pathophysiologic correlates that are responsible for the similar ECV values in DCM and HCM are not fully understood, but since DCM and HCM can usually be distinguished by their distinct ventricular geometry the overlap in ECV is clinically irrelevant. Furthermore, ECV elevation is typically pronounced in the mid-wall sections in DCM compared with RV hinge points and hypertrophied segments in HCM.

### Heart failure and heart failure with preserved ejection fraction

Heart failure is the final common pathway of many cardiomyopathies. Myocardial fibrosis – regardless of the aetiology – is a key mechanism in the development of diastolic and systolic heart failure. Since collagen deposition is often diffuse, LGE usually shows no regional fibrosis/scarring. According to data from the OPTIMIZE-HF registry preserved ejection fraction was present in a large proportion of patients with heart failure. Both heart failure patients with reduced and preserved ejection fraction experienced similar rates of mortality and morbidity [52]. Su et al. have shown that patients with systolic heart failure and heart failure with preserved ejection fraction (HFpEF) had elevated ECV in comparison with normal control subjects (31.2% vs. 28.9% vs. 27.9%) [53] (Fig. 6c). ECV elevation thus might help in identifying patients with worse prognosis otherwise undetected by conventional LGE techniques.

## Conclusions

Tissue characterisation by native T1 mapping may serve as an important source of diagnostic, therapeutic and prognostic decision making in various cardiac diseases.

An advantage of a non-invasive method for the assessment of fibrosis is the potential to follow changes in the myocardium over time as in patients with cardiomyopathies or patients receiving cardiotoxic drugs. Patients with poor renal function (or on dialysis) precluding gadolinium-based contrast injection may benefit from using native T1 mapping instead of LGE imaging. A clinical scenario where multi-parametric CMR tissue characterisation has already been established is in the assessment of patients with acute chest pain and no coronary artery disease. In such cases, tissue characterisation can assist in the differential diagnosis of micro-infarction, (peri-) myocarditis and stress cardiomyopathy (Takotsubo) and also other causes of diffuse fibrosis associated with high cardiac biomarker levels (such as high-sensitivity cardiac troponin).

Clinically, several studies have shown that T1 mapping with ECV is particularly useful in the assessment of cardiac diseases with diffuse fibrosis. Furthermore, T1 mapping with ECV might be helpful as an adjunct in cases with ambiguous LGE. Beyond differential diagnosis of cardiomyopathies, tissue characterization with T1 mapping can be very useful in differentiating between pericardial fat vs. LGE, differentiating between epicardial fat vs. pericardial effusion as well as in tissue characterization of various cardiac tumours.

More research is needed regarding the long-term prognostic impact of T1/ECV mapping, as well as its potential in therapy guidance of cardiac diseases such as heart failure, patients after heart transplantation as well as its role in valvular heart disease.

Harmonization of acquisition protocols between vendors and institution will also be needed to allow wider adoption of the methods.

Although tissue characterisation with native T1 and ECV has been shown to have incremental diagnostic benefit even in very early disease stages (e.g. diffuse fibrosis not detectable by LGE), there is an overlap between different cardiomyopathies and some overlap with normal T1 values. Like all medical parameters, abnormalities in native T1 and ECV need to be interpreted within their clinical context and pre-test probabilities and in conjunction with established CMR techniques such as LGE. Elevations and reductions of T1 and ECV are not specific and can be caused by various disease processes. In some instances, these processes can even cancel each other out (e.g. pseudonormalization in Anderson-Fabry disease when replacement fibrosis exceeds the fatty-related T1 decrease).

There is still some way to go with standardization of T1 mapping methods and protocols. Ongoing research for this purpose includes the use of standardized phantoms and software methods. For now, normal and pathological T1 values will largely depend on the acquisition scheme and will have to be defined in individual CMR centres.

## Abbreviations

AFD: Anderson-Fabry disease
AL: Light chains cardiac amyloidosis
ATTR: Transthyretin-related cardiac amyloidosis
CMR: Cardiovascular magnetic resonance
DCM: Dilated cardiomyopathy
ECV: Extracellular volume
HCM: Hypertrophic cardiomyopathy
HFpEF: Heart failure with preserved ejection fraction
LGE: Late gadolinium enhancement
LV: Left ventricular
MOLLI: Modified look-locker inversion recovery
RA: Rheumatoid arthritis
ROI: Region of interest
SAPPHIRE: Saturation pulse prepared heart-rate-independent inversion recovery
SASHA: Saturation recovery single-shot acquisition
ShMOLLI: Shortened modified look-locker inversion recovery
SI: Signal intensity
STIR: Short-tau inversion recovery
T1: Longitudinal recovery time
